# TRPV4 activation by core body temperature has multimodal functions in the central nervous system

**DOI:** 10.1186/s12576-024-00948-x

**Published:** 2024-11-22

**Authors:** Koji Shibasaki

**Affiliations:** Laboratory of Neurochemistry, Department of Nutrition Science, University of Nagasaki, Nagasaki, 851-2195 Japan

**Keywords:** TRPV4, Thermosensor, Brain, Temperature

## Abstract

Brain temperature is strictly regulated by various endogenous mechanisms and significantly contributes to brain function in homeothermic animals, making it an important factor for health. Thermosensitive transient receptor potential (TRP) channels convert temperature information into electrical signals through cation influx. In particular, TRPV4 is involved in the regulation of brain function. TRPV4, constitutively active in neurons through its activation by brain temperature, increases neuronal firing. TRPV4KO mice have electroencephalogram abnormalities, resulting in depression-like and social behavioral abnormalities. This basic function of TRPV4, as a translator of brain temperature information, has been implicated in several diseases, including epilepsy and stress-induced depression. In addition to its neuronal functions, TRPV4 has many key functions in glia and vasculature that depend on brain temperature and contribute to brain activity. In this review, I summarize the importance of TRPV4 activities in relation to brain temperature and focus on how hyperthermia-induced TRPV4 dysfunction exacerbates brain diseases.

## Introduction

Transient receptor potential (TRP) channels are present in many different species [1, 2]. Most TRP channels function as polymodal receptors. Some expressed in nociceptive sensory neurons mediate pain [3], making them suitable targets for the treatment of acute and chronic pain [3–5]. In particular, recent progress in TRPV1 research has been significant and impressive, as the discovery of TRPV1 was awarded the 2021 Nobel Prize in Physiology or Medicine. The mammalian TRP channel superfamily is classified into six subfamilies [1], which are TRPV (vanilloid), TRPA (ANKTM1), TRPC (canonical), TRPM (melastatin), TRPML (mucolipin), and TRPP (polycystin) [3]. In this review, I summarize the physiological importance of TRPV4 as a multimodal receptor for specific brain functions related to core body temperature.

### TRPV4 ion channel

Originally, TRPV4 was identified as an osmotically activated channel [6]. Consistent with this characteristic, TRPV4 can be activated by membrane stretch [6, 7]. Liedtke et al. reported that warmer temperatures also activated TRPV4 [6]. The mechanism by which cellular swelling activates TRPV4 relates to activation of phospholipase A_2_ (PLA_2_) [8, 9]. Hydrolysis of membrane lipids by PLA_2_ produces arachidonic acid (AA), and AA is metabolized to epoxyeicosatrienoic acids (EETs) by cytochrome P450. EETs can activate the TRPV4 channel directly [9, 10]. Since TRPV4 is a thermosensitive TRP, it was later determined as a thermosensor activated by temperatures in the range of 27 ~ 34 °C [11, 12]. The temperature thresholds of TRPV4 have been the subject of debate. When TRPV4 was first cloned, it was shown that rat TRPV4 was activated at near core body temperature in CHOK1 cells [6]. In addition, it was reported that rat TRPV4 is activated by physiological temperature (> 34 °C) in HEK cells [12]. In contrast, it was also reported that mouse TRPV4 was activated by temperatures above 27 °C in HEK cells [11]. Other groups reported that mouse TRPV4 was activated by physiological temperatures (> 34 °C) in naïve cells [5, 13]. These differences might arise from the content of AA or EETs in the various experimental conditions. In fact, heat activation of TRPV4 requires endogenous ligands. It was reported that inside–out membrane patches in TRPV4-expressing cells failed to produce heat-evoked currents [11], indicating that heat activation of TRPV4 relies on intracellular ligands.

### Brain temperature maintains brain function through TRPV4 activation

**Fig. 1:**
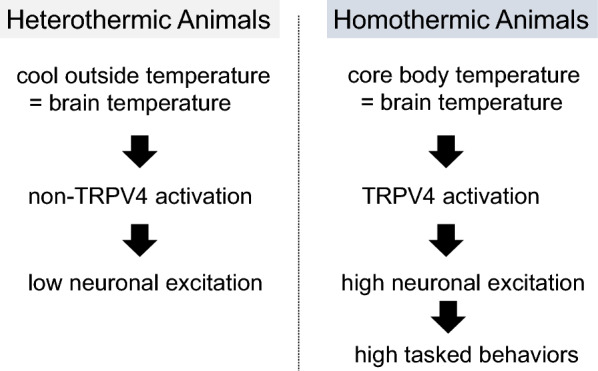
Background of high-tasked behaviors in homeothermic animals

Shibasaki et al. found that TRPV4 is abundantly expressed in hippocampal neurons [13]. Specifically, neuronal TRPV4 is localized post-synaptically by a regulated synaptic targeting pathway [14]. TRPV4 is constitutively active in hippocampal neurons through its activation by brain temperature and enhances neuronal firing [13, 15]. Similar to our finding, it has been reported that TRPV4 activation in neurons of the substantia nigra pars compacta increases the frequency of neuronal firing. It is considered that this basic neuronal property naturally enhances brain activity. Indeed, Shibasaki et al. also found that theta-frequency electroencephalogram (EEG) activities in TRPV4-deficient (TRPV4KO) mice during wake periods were significantly reduced compared with those in wild type (WT) mice [15]. The reduced brain activity in TRPV4KO mice seemed to affect their behavior. It was also found that TRPV4KO mice exhibited reduced depression-like and social behaviors compared with WT mice [15]. Genome-wide association studies between control subjects and subjects with major depressive disorder revealed that TRPV4 mutations are a risk factor for depression [16]. Indeed, whole-genome sequencing of 85 quartet families with autism spectrum disorder (ASD) revealed that frameshift mutations in *TRPV4* were present in the family members with ASD [17]. These studies indicate that disruption of TRPV4 function relates to psychiatric symptoms. Thus, detecting brain temperature through TRPV4 activation finely enhances neuronal firing (probably together with other thermosensitive channels activated by brain temperature) and contributes to synchronize neuronal excitations. Shibasaki et al. have established a new concept that neuronal TRPV4 senses brain temperature and contributes to neuronal excitability and behaviors in mammals (Fig. 1). Based on the temperature threshold of TRPV4 (> 34 °C) in the hippocampal neurons related to the data [13, 15], it is hypothesized that these observations about the contribution of TRPV4 to neuronal excitability and behavior might also apply to homeothermic animals in general. Compared with heterothermic animals, birds and mammals must perform many high-tasked behaviors, such as constructing shelter and nurturing offspring (Fig. 1). In addition to differences in brain structure among different species, our ancestors evolved specific characteristics to maintain body temperature as homeothermic animals. Thus, the brain has acquired effective mechanisms to convert thermal information into neuronal excitability at any given time. These features might accelerate species-specific behaviors in birds and mammals, and TRPV4 might be important in these evolutionary steps as a major factor contributing to brain function.

**Fig. 2:**
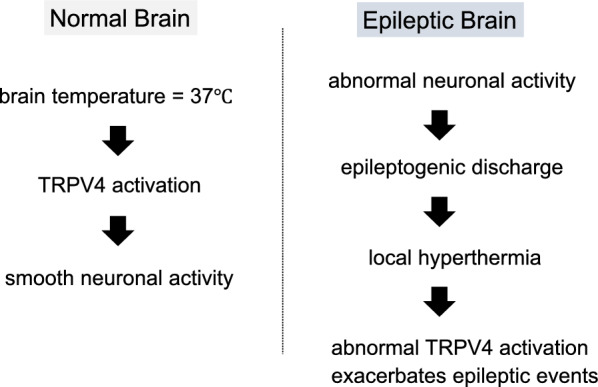
Epileptogenic discharges evoke local hyperthermia and hyperthermia-induced abnormal TRPV4 activation

For many years, brain cooling was thought to be an effective way of suppressing epileptic discharges; however, the underlying molecular mechanisms were unknown [18, 19]. Shibasaki et al. have revealed that epilepsy elevated brain temperature about 1 °C in epileptogenic foci, and the heat accelerates abnormal TRPV4 activation (Fig. 2) [20, 21]. Since TRPV4 activation (by brain temperature) contributes to brain activity, abnormal TRPV4 activation exacerbates epileptic discharges. Thus, brain cooling can inhibit abnormal TRPV4 activation. Indeed, the suppressive effect of cooling on epileptic discharges in TRPV4KOs was significantly smaller than that in WT mice [20]. The results indicate that brain cooling inactivates TRPV4 to suppress epileptic discharges. Alternatively, the injection of a TRPV4-specific inhibitor also effectively suppressed discharges. We revealed molecular mechanisms contributing to the efficacy of brain cooling at epileptogenic foci to effectively suppress epileptic discharge.

It was also shown that heat-induced abnormal TRPV4 activation exacerbated brain edema [22]. Brain edema is characterized by excessive water accumulation, resulting in an increase in brain volume. Brain edema is commonly associated with ischemic stroke with severe middle cerebral artery or hemispheric infarctions [23]. Hoshi et al. found that in mouse brain slices, oxygen–glucose deprivation in the first layer of the parietal association cortex initiated brain temperature elevation (about 2 °C range) through glutamate receptor activation and induced abnormal TRPV4 activation. TRPV4 activation is enhanced by heating and additively activated in response to an increase in temperature. Therefore, heat (2 °C)-evoked TRPV4 sensitization in specific temperature-elevated regions may further progress the development of brain edema through an additive TRPV4 activation and may also be relevant for excitotoxic injury in neurodegeneration [22]. These results indicate that TRPV4 is a key determinant in the induction of brain edema after ischemia-induced hyperthermia. In addition, TRPV4 activation is sufficient to induce brain edema. We have also reported that TRPV4 activation enhanced NMDA receptor activation [13]. Therefore, TRPV4 activation is involved in multiple different steps, such as enhancement of NMDA receptor activation and Ca^2+^ influx by hyperthermia-induced activation, resulting in dysregulation of cell volume changes during the process of brain edema.

Microglia are resident immune cells in the central nervous system. Microglia maintain brain homeostasis by monitoring changes in the environment in their resting state [24]. Activated microglia can have protective functions by responding to environmental changes. Both surveillance and protective roles require microglial contact movement, a key action of both roles. This dynamic process is affected by the surrounding environment [25]. Professor Tominaga’s group, for the first time, discovered that microglial movement is temperature dependent [26]. This temperature dependency relies mainly on TRPV4 activity. Thus, TRPV4 is a strong modulator for microglial functions depending on brain temperature fluctuations and might be involved in CNS pathologies. Indeed, it has been reported that microglial-induced neuronal damage is caused via the TRPV4–AMP-activated protein kinase–NF-κB pathway and that therapeutic hypothermia can suppress microglial activation through inactivation of the TRPV4 pathway [27]. In addition, robust microglial activation after an injection of lipopolysaccharide into the mouse cerebral ventricle was suppressed by the activation of TRPV4 [28]. The release of tumor necrosis factor-α (TNF-α) and expression of galectin-3 were both increased by lipopolysaccharide; however, those increases were significantly suppressed by the activation of TRPV4. The amplitude of voltage-dependent K^+^ current was also suppressed by the activation of TRPV4. Opening of TRPV4 channels induced membrane depolarization mainly by increasing Na^+^ influx in microglia [28]. These results indicate that TRPV4 activation in microglia has a protective effect to suppress the inflammatory state of the cells.

Several *TRPV4* mutations cause peripheral neuropathy. Mutations in *TRPV4* are associated with many severe diseases, such as scapuloperoneal spinal muscular atrophy, Charcot–Marie–Tooth disease type 2C (CMT2C) and skeletal dysplasia [29, 30]. To investigate the effects of CMT2C-associated *TRPV4* mutations, WT and mutant TRPV4 were expressed in *Xenopus laevis* oocytes. Oocytes expressing the R269C and R269H mutants showed a 2.5-fold increase in TRPV4 currents compared with WT under resting conditions (at 18 °C). Both mutants showed a five fold increase in TRPV4 currents compared with WT at physiological temperature (38 °C), indicating that both mutants are abnormal constitutive active forms at body temperature [31]. These observations are consistent with the findings in the TRPV4 cloning report, which described that modulation of TRPV4 activity with temperature adapted to core body temperature [6]. Thus, TRPV4 gain-of-function mutations are responsible for CMT2C disease progression.

### Social stress induces abnormal hippocampal TRPV4 activation and results in stress-induced depression

Social stress significantly impairs neurogenesis in the hippocampal dentate gyrus, resulting in maladaptive behaviors [32, 33]. Social stress has been shown to induce core hyperthermia as psychogenic fever, and this hyperthermia might relate to the maladaptive behaviors [34, 35]. To address this possibility, we investigated the involvement of TRPV4 in impairment of hippocampal neurogenesis. Hoshi et al. analyzed a mouse model of social defeat stress. Social defeat stress significantly induced psychogenic fever. The investigators detected a 2 °C elevation of core body temperature after social defeat stress in the mouse model [36]. It was hypothesized that the hyperthermia induced abnormal TRPV4 activation and caused maladaptive behaviors, and it was found that hippocampal neural stem cells (NSCs) expressed a very high level of TRPV4 in the subgranular zone of the dentate gyrus [36]. Social defeat stress induced abnormal TRPV4 activation through hyperthermia and reduced the number of NSCs in a TRPV4 activation-dependent manner. Abnormal TRPV4 activation by hyperthermia triggers externalization of phosphatidylserine on NSCs, which is recognized by microglia as an “eat-me” signal. Thus, microglia abnormally engulf the phosphatidylserine-externalized NSCs after social defeat stress, which results in a significant reduction in NSC numbers [36]. It was demonstrated that the reduction in NSC numbers by TRPV4 activation was responsible for the induction of stress-induced depression in rodents. Thus, the inhibition of abnormal activation of TRPV4 in NSCs perfectly abolished the stress-induced depression [36]. Taken together, these findings revealed that TRPV4 is the most suitable therapeutic target for stress-induced depression.

### TRPV4 contributes to cerebrospinal fluid production

Choroid plexus epithelial cells (CPECs) have the highest level of TRPV4 expression in the brain. In CPECs, TRPV4 plays a pivotal role in the calcium homeostasis of cerebrospinal fluid (CSF) through α-klotho type 1 [37]. The choroid plexus contributes to maintenance of the brain environment in the lateral, third, and fourth ventricles, and makes a specific unit as the blood–CSF barrier. The most important function of CPECs is CSF production, which is dependent on electrolyte transport from the basolateral to apical membranes of the CPECs and involves many ion transport proteins [38]. Takayama et al. found that TRPV4 in CPECs is activated by brain temperature, and CPEC movement further activates TRPV4 through the production of EETs by mechanical stimuli. TRPV4 activation leads to the activation of anoctamin 1 (ANO1)/TMEM16A, a Ca^2+^-activated chloride channel, and causes significant Cl^−^ efflux. This Cl^−^ efflux can be a specific driving force for water efflux from intracellular to extracellular spaces in CPECs [38]. Hence, the choroid plexus can effectively generate a large amount of CSF without ineffectual energy loss. Another study revealed a different function of TRPV4 in the choroid plexus; the activation of TRPV4 induced a marked decrease in filamentous actin, disintegrated the cell junctions, and affected basolateral-to-apical transport [39]. These studies indicate that TRPV4 produces the specific water movement-driving force for CSF production.

### TRPV4 regulates vascular tone

Vascular tone plays a pivotal role in regulating blood flow in animal tissues. Control of the contraction of vascular smooth muscle cells (VSMCs) in the systemic circulation relies on vasoconstrictor and vasodilator stimulation by circulating substances, such as neurotransmitters, hormones, and endothelial cell (EC)-derived factors. Membranous ion channels can detect various stimuli in both ECs and VSMCs and affect cellular responses, including vascular contraction and relaxation [40–42]. Both ECs and VSMCs express TRPV4, including those in the brain [40]. As described above, TRPV4 is constitutively activated by core body temperature [15]. TRPV4 has unique properties as synergistic effects. Therefore, when TRPV4 senses two different agonists, the thresholds of each agonist can be effectively reduced. As a result of the reductions in thresholds, we can observe significant TRP channel activation with a combination of two different agonists [21]. Thus, TRPV4 can be constitutively potentiated by brain temperature and play a role as a base for synergistic effects. It was demonstrated that TRPV4 sensed shear stress and contributed to the vasodilation of arteries [43, 44]. In this case, brain temperature significantly elevated TRPV4 sensation of shear stress. It was also reported that shear stress increases the sensitivity of TRPV4 agonists in ECs and has a relationship between TRPV4 activation and muscarinic receptor-induced endothelium-dependent relaxation. Nitric oxide (NO) induces endothelium-dependent relaxation in large arteries [45]. It was also reported that TRPV4 has a special link with NO generation and NO-related relaxation [46, 47]. Shear stress and acetylcholine increase TRPV4-induced Ca^2+^ influx and activate endothelial NO synthase (eNOS) through its phosphorylation in ECs [48]. Then, ECs release NO, and the NO triggers arterial dilation.

VSMCs have a pivotal function in vasocontraction [49–51]. TRPV4 is expressed in VSMCs as well as in ECs [40, 52]. 5-Hydrixytryptamine induces TRPV4 activation through Gq-coupled signaling and causes vascular contraction. Phenylephrine or endothelin-1 can also induce vascular contraction through VSMC TRPV4 activation [53]. Specific communication between ECs and VSMCs also plays an important role for vascular tone regulation, and TRPV4 contributes to this trans-cellular communication. VSMC TRPV4 is a critical determinant for vascular tone control.

### TRPV4 in astrocytes and oligodendrocyte precursor cells

Shibasaki et al. found that TRPV4 expression was restricted to ~ 30% of the specific astrocyte population, indicating that astrocytic subtypes can be classified based on their expression patterns [54]. When TRPV4-positive astrocytes are activated by endogenous ligands such as arachidonic acid and its derivatives, the activation propagates to neighboring astrocytes through gap junctions and by ATP release from the TRPV4-positive astrocytes. Following activation, both TRPV4-positive and TRPV4-negative astrocytes release glutamate to increase synaptic transmission through group 1 mGluR [54]. These results indicate that TRPV4-positive astrocytes constitute a novel subtype of the population and are solely responsible for initiating excitatory gliotransmitter release to enhance synaptic transmission. In this case, astrocytic TRPV4 can be constitutively potentiated by brain temperature and play a role as a base for synergistic effects, as described above. TRPV4 is mainly localized at the endfeet of astrocytes, where it regulates the blood brain barrier [55]. In particular, astrocytic TRPV4 can modulate neurovascular coupling and regulate brain blood flow. Astrocytic TRPV4 has been identified to be involved in Ca^2+^-induced processes in ischemic excitotoxicity, peri-infarct depolarization, and oxidative stress or astrocytic swelling [56]. Thus, astrocytic TRPV4 is a good therapeutic target for brain ischemia.

It is reported that oligodendrocyte precursor cells (OPCs) and mature oligodendrocytes express TRPV4 [56]; however, its functions remain to be elucidated. Ohashi et al. reported that TRPV4 is functionally expressed in OPCs, and TRPV4 activation increases the proliferation of these cells without affecting their ability to differentiate into oligodendrocytes [57]. These results suggest that the proliferation rate of OPCs in homeothermic animals might be faster than in heterothermic ones, since constant core body temperature can constitutively activate TRPV4 in OPCs. Thus, birds and mammals might have specific advantages to maintain much larger numbers of OPCs compared with heterothermic animals by utilizing brain temperature. TRPV4 is also functionally expressed in non-myelinating Schwann cells [58, 59]. TRPV4 expression in non-myelinating Schwann cells is increased in demyelinating sciatic nerves after injury treatment [60]. Loss of TRPV4 impairs functional recovery and remyelination of sciatic nerves, indicating that TRPV4 in non-myelinating Schwann cells has a specific function for myelin repair [60].

### Core body temperature significantly affects neuronal disease through synergistic activation of TRPV4

**Fig. 3:**
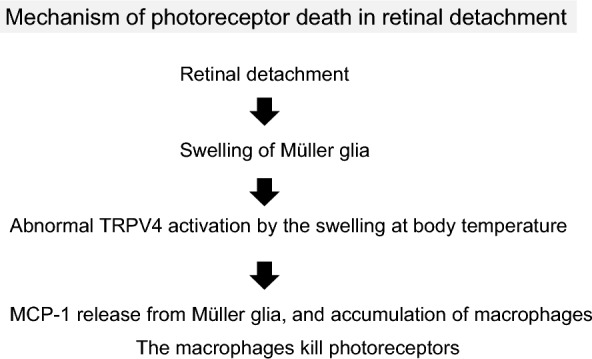
Mechanism of photoreceptor death in retinal detachment

TRPV4 can act as a heat and/or stretch sensor. Notably, weak artificial mechanical stimuli did not activate any mechano-sensing activity in TRPV4-expressing HEK293 cells. In contrast, significant TRPV4 mechano-sensitivities were observed in retinal Müller glia [61]. These results indicate that naïve cells, such as Müller glia, possess specific characteristics that enhance the TRPV4 mechano-sensitivities. As described above, based on our data, TRPV4 is activated at temperatures above 34 °C [13], and various ligands combined with increased temperature have synergistic-potentiation effects on TRVP4 activation [21]. It is hypothesized that body temperature significantly reduces the TRPV4-activation threshold against mechanical stimuli. Therefore, Matsumoto et al. performed whole-cell patch clamp recordings by application of computer-programmed mechanical stimuli at different temperatures. They applied minimal artificial membrane stretch (10 mmHg) to Müller glia at 25 °C or 37 °C and recorded the mechanical stimuli-evoked TRPV4 currents at each temperature. Compared with 25 °C conditions, physiological temperature (37 °C) significantly potentiated the mechano-sensing activity of TRPV4 [61]. These results indicate that body temperature significantly elevates TRPV4 sensitivity to membrane stretch as a result of the synergistic effect of temperature and mechanical stimuli. In retinal detachment, significant Müller glial swelling has been observed in reactive glia [62, 63]. Taken together, these findings suggest that retinal detachment-induced Müller glial swelling significantly activates TRPV4 channels. Furthermore, they found that this swelling-induced TRPV4 activation triggered Ca^2+^ influx and evoked MCP-1 release in Müller glia. Excessive amounts of MCP-1 recruit numerous macrophages near the retina, which target and kill photoreceptors (Fig. 3) [61]. In addition to hypoxia, this is the critical mechanism by which photoreceptor death is exacerbated in retinal detachment. Thus, TRPV4 inhibitors could suppress photoreceptor death in retinal detachment, and targeting TRPV4 in Müller glial might represent a novel therapeutic target for the prevention of photoreceptor cell death after retinal detachment.

## Conclusion

The maintenance of brain temperature is very important for health. Brain temperature is a critical determinant for neuronal excitation. Hence, acute hypothermia causes severe shivering, lethargy and reduction of heart function. TRPV4 senses body temperature and, more specifically, TRPV4 channels detect dynamic local temperature changes in the brain. It is well known that therapeutic hypothermia is effective for several diseases, such as ischemic stroke, traumatic brain injury and cardiac arrest, due to its ability to reduce oxygen consumption, cellular inflammation and blood flow. In contrast to hypothermia, hyperthermia over 43 °C induces severe cell death. TRPV4 inactivation or activation is involved in these therapeutic interventions. Constitutive activation of TRPV4 by brain temperature is necessary for various brain functions; however, strict control of TRPV4 is very important for therapeutic hypothermia. Future studies will reveal further important functions of TRPV4 activation by brain temperature.

We can consider that the contribution of TRPV4 to neuronal excitability and behaviors applies to homeothermic animals. Compared with heterothermic animals, birds and mammals must perform many high-tasked behaviors, such as constructing shelter and nurturing offspring. In addition to differences in brain structure among different species, our ancestors evolved specific characteristics to maintain body temperature as homeothermic animals. TRPV4 can be constitutively active in the brains of homeothermic animals, different from heterothermic animals. Thus, the brains of homeothermic animals have acquired effective mechanisms to convert thermal information to neuronal excitability at any given time. In heterothermic animals, one possibility is that they can activate TRPV4 as needed, while homeothermic animals have constitutively activated TRPV4. On cool days, we often see lizards resting on hot stones to increase their body temperature. The increased temperature can activate their brain TRPV4. After these changes occur, they can maximize their predatory behavior.

Shibasaki et al. have demonstrated that epilepsy elevated brain temperature about 1 °C in epileptogenic foci, and the local hyperthermia accelerated abnormal TRPV4 activation. The abnormal TRPV4 activation exacerbates epileptic discharges.

Retinal detachment-induced Müller glial swelling significantly activates TRPV4 channels at body temperature. TRPV4 is synergistically activated by two different stimuli, swelling and body temperature. Furthermore, this swelling-induced TRPV4 activation triggered Ca^2+^ influx and evoked MCP-1 release from Müller glia. Excessive amounts of MCP-1 recruit numerous macrophages near the retina to target and kill photoreceptors.

## Data Availability

The data that support the findings of this manuscript are available from the corresponding author, K.S., upon reasonable request.

## References

[1] Shibasaki K (2016) Physiological significance of TRPV2 as a mechanosensor, thermosensor and lipid sensor. J Physiol Sci 66:359–365 26841959 10.1007/s12576-016-0434-7 PMC10717341

[2] Clapham DE (2003) TRP channels as cellular sensors. Nature 426:517–524 14654832 10.1038/nature02196

[3] Tominaga M, Caterina MJ (2004) Thermosensation and pain. J Neurobiol 61:3–12 15362149 10.1002/neu.20079

[4] Levine JD, Alessandri-Haber N (2007) TRP channels: targets for the relief of pain. Biochim Biophys Acta 1772:989–1003 17321113 10.1016/j.bbadis.2007.01.008

[5] Chung MK, Lee H, Caterina MJ (2003) Warm temperatures activate TRPV4 in mouse 308 keratinocytes. J Biol Chem 278:32037–32046 12783886 10.1074/jbc.M303251200

[6] Liedtke W, Choe Y, Marti-Renom MA, Bell AM, Denis CS, Sali A, Hudspeth AJ, Friedman JM, Heller S (2000) Vanilloid receptor-related osmotically activated channel (VR-OAC), a candidate vertebrate osmoreceptor. Cell 103:525–535 11081638 10.1016/s0092-8674(00)00143-4 PMC2211528

[7] Strotmann R, Harteneck C, Nunnenmacher K, Schultz G, Plant TD (2000) OTRPC4, a nonselective cation channel that confers sensitivity to extracellular osmolarity. Nat Cell Biol 2:695–702 11025659 10.1038/35036318

[8] Goldenberg NM, Ravindran K, Kuebler WM (2015) TRPV4: physiological role and therapeutic potential in respiratory diseases. Naunyn Schmiedebergs Arch Pharmacol 388:421–436 25342095 10.1007/s00210-014-1058-1

[9] Ryskamp DA, Jo AO, Frye AM, Vazquez-Chona F, MacAulay N, Thoreson WB, Krizaj D (2014) Swelling and eicosanoid metabolites differentially gate TRPV4 channels in retinal neurons and glia. J Neurosci 34:15689–15700 25411497 10.1523/JNEUROSCI.2540-14.2014 PMC4236400

[10] Watanabe H, Vriens J, Prenen J, Droogmans G, Voets T, Nilius B (2003) Anandamide and arachidonic acid use epoxyeicosatrienoic acids to activate TRPV4 channels. Nature 424:434–438 12879072 10.1038/nature01807

[11] Watanabe H, Vriens J, Suh SH, Benham CD, Droogmans G, Nilius B (2002) Heat-evoked activation of TRPV4 channels in a HEK293 cell expression system and in native mouse aorta endothelial cells. J Biol Chem 277:47044–47051 12354759 10.1074/jbc.M208277200

[12] Guler AD, Lee H, Iida T, Shimizu I, Tominaga M, Caterina M (2002) Heat-evoked activation of the ion channel, TRPV4. J Neurosci 22:6408–6414 12151520 10.1523/JNEUROSCI.22-15-06408.2002 PMC6758176

[13] Shibasaki K, Suzuki M, Mizuno A, Tominaga M (2007) Effects of body temperature on neural activity in the hippocampus: regulation of resting membrane potentials by transient receptor potential vanilloid 4. J Neurosci 27:1566–1575 17301165 10.1523/JNEUROSCI.4284-06.2007 PMC6673744

[14] Shibasaki K, Tominaga M, Ishizaki Y (2015) Hippocampal neuronal maturation triggers post-synaptic clustering of brain temperature-sensor TRPV4. Biochem Biophys Res Commun 458:168–173 25637662 10.1016/j.bbrc.2015.01.087

[15] Shibasaki K, Sugio S, Takao K, Yamanaka A, Miyakawa T, Tominaga M, Ishizaki Y (2015) TRPV4 activation at the physiological temperature is a critical determinant of neuronal excitability and behavior. Pflugers Arch 467:2495–2507 26250433 10.1007/s00424-015-1726-0

[16] Wong ML, Arcos-Burgos M, Liu S, Velez JI, Yu C, Baune BT, Jawahar MC, Arolt V, Dannlowski U, Chuah A, Huttley GA, Fogarty R, Lewis MD, Bornstein SR, Licinio J (2017) The PHF21B gene is associated with major depression and modulates the stress response. Mol Psychiatry 22:1015–1025 27777418 10.1038/mp.2016.174 PMC5461220

[17] Yuen RK, Thiruvahindrapuram B, Merico D, Walker S, Tammimies K, Hoang N, Chrysler C, Nalpathamkalam T, Pellecchia G, Liu Y, Gazzellone MJ, D’Abate L, Deneault E, Howe JL, Liu RS, Thompson A, Zarrei M, Uddin M, Marshall CR, Ring RH, Zwaigenbaum L, Ray PN, Weksberg R, Carter MT, Fernandez BA, Roberts W, Szatmari P, Scherer SW (2015) Whole-genome sequencing of quartet families with autism spectrum disorder. Nat Med 21:185–191 25621899 10.1038/nm.3792

[18] Baldwin M, Frost LL (1956) Effect of hypothermia on epileptiform activity in the primate temporal lobe. Science 124:931–932 13380404 10.1126/science.124.3228.931-a

[19] Karlov VA (2003) Focal cooling suppresses continued activity of epileptic focus in patients with partial status epilepticus. Epilepsia 44:1605 14636337 10.1111/j.0013-9580.2003.33003.x

[20] Shibasaki K, Yamada K, Miwa H, Yanagawa Y, Suzuki M, Tominaga M, Ishizaki Y (2020) Temperature elevation in epileptogenic foci exacerbates epileptic discharge through TRPV4 activation. Lab Invest 100:274–284 31641226 10.1038/s41374-019-0335-5

[21] Shibasaki K (2020) TRPV4 activation by thermal and mechanical stimuli in disease progression. Lab Invest 100:218–223 31896814 10.1038/s41374-019-0362-2

[22] Hoshi Y, Okabe K, Shibasaki K, Funatsu T, Matsuki N, Ikegaya Y, Koyama R (2018) Ischemic brain injury leads to brain edema via hyperthermia-induced TRPV4 activation. J Neurosci 38:5700–5709 29793978 10.1523/JNEUROSCI.2888-17.2018 PMC6595977

[23] Aiyagari V, Diringer MN (2002) Management of large hemispheric strokes in the neurological intensive care unit. Neurologist 8:152–162 12803687 10.1097/00127893-200205000-00002

[24] Wolf SA, Boddeke HW, Kettenmann H (2017) Microglia in physiology and disease. Annu Rev Physiol 79:619–643 27959620 10.1146/annurev-physiol-022516-034406

[25] Nimmerjahn A, Kirchhoff F, Helmchen F (2005) Resting microglial cells are highly dynamic surveillants of brain parenchyma in vivo. Science 308:1314–1318 15831717 10.1126/science.1110647

[26] Nishimoto R, Derouiche S, Eto K, Deveci A, Kashio M, Kimori Y, Matsuoka Y, Morimatsu H, Nabekura J, Tominaga M (2021) Thermosensitive TRPV4 channels mediate temperature-dependent microglia movement. Proc Natl Acad Sci U S A. 10.1073/pnas.2012894118 33888579 10.1073/pnas.2012894118 PMC8092382

[27] Fukuda N, Toriuchi K, Mimoto R, Aoki H, Kakita H, Suzuki Y, Takeshita S, Tamura T, Yamamura H, Inoue Y, Hayashi H, Yamada Y, Aoyama M (2024) Hypothermia attenuates neurotoxic microglial activation via TRPV4. Neurochem Res 49:800–813 38112974 10.1007/s11064-023-04075-8

[28] Konno M, Shirakawa H, Iida S, Sakimoto S, Matsutani I, Miyake T, Kageyama K, Nakagawa T, Shibasaki K, Kaneko S (2012) Stimulation of transient receptor potential vanilloid 4 channel suppresses abnormal activation of microglia induced by lipopolysaccharide. Glia 60:761–770 22331560 10.1002/glia.22306

[29] Sullivan JM, Bagnell AM, Alevy J, Avila EM, Mihaljevic L, Saavedra-Rivera PC, Kong L, Huh JS, McCray BA, Aisenberg WH, Zuberi AR, Bogdanik L, Lutz CM, Qiu Z, Quinlan KA, Searson PC, Sumner CJ (2024) Gain-of-function mutations of TRPV4 acting in endothelial cells drive blood-CNS barrier breakdown and motor neuron degeneration in mice. Sci Transl Med 16:eadk1358 38776392 10.1126/scitranslmed.adk1358 PMC11316273

[30] Berth SH, Vo L, Kwon DH, Grider T, Damayanti YS, Kosmanopoulos G, Fox A, Lau AR, Carr P, Donohue JK, Hoke M, Thomas S, Karim C, Fay AJ, Meltzer E, Crawford TO, Gaudet R, Shy ME, Hellmich UA, Lee SY, Sumner CJ, McCray BA (2024) Combined clinical, structural, and cellular studies discriminate pathogenic and benign TRPV4 variants. Brain. 10.1093/brain/awae243 39021275 10.1093/brain/awae243 PMC12054728

[31] Landoure G, Zdebik AA, Martinez TL, Burnett BG, Stanescu HC, Inada H, Shi Y, Taye AA, Kong L, Munns CH, Choo SS, Phelps CB, Paudel R, Houlden H, Ludlow CL, Caterina MJ, Gaudet R, Kleta R, Fischbeck KH, Sumner CJ (2010) Mutations in TRPV4 cause Charcot-Marie-Tooth disease type 2C. Nat Genet 42:170–174 20037586 10.1038/ng.512 PMC2812627

[32] Miller BR, Hen R (2015) The current state of the neurogenic theory of depression and anxiety. Curr Opin Neurobiol 30:51–58 25240202 10.1016/j.conb.2014.08.012 PMC4293252

[33] Denoth-Lippuner A, Jessberger S (2021) Formation and integration of new neurons in the adult hippocampus. Nat Rev Neurosci 22:223–236 33633402 10.1038/s41583-021-00433-z

[34] Machado NLS, Abbott SBG, Resch JM, Zhu L, Arrigoni E, Lowell BB, Fuller PM, Fontes MAP, Saper CB (2018) A glutamatergic hypothalamomedullary circuit mediates thermogenesis, but not heat conservation, during stress-induced hyperthermia. Curr Biol 28(2291–2301):e2295 10.1016/j.cub.2018.05.064 PMC6085892 30017482

[35] Kataoka N, Shima Y, Nakajima K, Nakamura K (2020) A central master driver of psychosocial stress responses in the rat. Science 367:1105–1112 32139538 10.1126/science.aaz4639

[36] Hoshi Y, Shibasaki K, Gailly P, Ikegaya Y, Koyama R (2021) Thermosensitive receptors in neural stem cells link stress-induced hyperthermia to impaired neurogenesis via microglial engulfment. Sci Adv 7:eabj8080 34826234 10.1126/sciadv.abj8080 PMC8626080

[37] Imura A, Tsuji Y, Murata M, Maeda R, Kubota K, Iwano A, Obuse C, Togashi K, Tominaga M, Kita N, Tomiyama K, Iijima J, Nabeshima Y, Fujioka M, Asato R, Tanaka S, Kojima K, Ito J, Nozaki K, Hashimoto N, Ito T, Nishio T, Uchiyama T, Fujimori T, Nabeshima Y (2007) alpha-Klotho as a regulator of calcium homeostasis. Science 316:1615–1618 17569864 10.1126/science.1135901

[38] Takayama Y, Shibasaki K, Suzuki Y, Yamanaka A, Tominaga M (2014) Modulation of water efflux through functional interaction between TRPV4 and TMEM16A/anoctamin 1. Faseb J 28:2238–2248 24509911 10.1096/fj.13-243436

[39] Narita K, Sasamoto S, Koizumi S, Okazaki S, Nakamura H, Inoue T, Takeda S (2015) TRPV4 regulates the integrity of the blood-cerebrospinal fluid barrier and modulates transepithelial protein transport. Faseb J 29:2247–2259 25681460 10.1096/fj.14-261396

[40] Matsumoto T, Taguchi K, Kobayashi T (2023) Role of TRPV4 on vascular tone regulation in pathophysiological states. Eur J Pharmacol 959:176104 37802278 10.1016/j.ejphar.2023.176104

[41] Goulopoulou S, Webb RC (2014) Symphony of vascular contraction: how smooth muscle cells lose harmony to signal increased vascular resistance in hypertension. Hypertension 63:e33-39 24470463 10.1161/HYPERTENSIONAHA.113.02444 PMC3966483

[42] Vanhoutte PM, Shimokawa H, Feletou M, Tang EH (2017) Endothelial dysfunction and vascular disease—a 30th anniversary update. Acta Physiol (Oxf) 219:22–96 26706498 10.1111/apha.12646

[43] Kohler R, Heyken WT, Heinau P, Schubert R, Si H, Kacik M, Busch C, Grgic I, Maier T, Hoyer J (2006) Evidence for a functional role of endothelial transient receptor potential V4 in shear stress-induced vasodilatation. Arterioscler Thromb Vasc Biol 26:1495–1502 16675722 10.1161/01.ATV.0000225698.36212.6a

[44] Liu L, Guo M, Lv X, Wang Z, Yang J, Li Y, Yu F, Wen X, Feng L, Zhou T (2021) Role of transient receptor potential vanilloid 4 in vascular function. Front Mol Biosci 8:677661 33981725 10.3389/fmolb.2021.677661 PMC8107436

[45] Brandes RP, Schmitz-Winnenthal FH, Feletou M, Godecke A, Huang PL, Vanhoutte PM, Fleming I, Busse R (2000) An endothelium-derived hyperpolarizing factor distinct from NO and prostacyclin is a major endothelium-dependent vasodilator in resistance vessels of wild-type and endothelial NO synthase knockout mice. Proc Natl Acad Sci U S A 97:9747–9752 10944233 10.1073/pnas.97.17.9747 PMC16936

[46] Mao A, Zhang P, Zhang K, Kan H, He D, Han X, Wang Z, Tang C, Ma X (2022) Endothelial TRPV4-eNOS coupling as a vital therapy target for treatment of hypertension. Br J Pharmacol 179:2297–2312 34822720 10.1111/bph.15755

[47] Marziano C, Hong K, Cope EL, Kotlikoff MI, Isakson BE, Sonkusare SK (2017) Nitric oxide-dependent feedback loop regulates transient receptor potential vanilloid 4 (TRPV4) channel cooperativity and endothelial function in small pulmonary arteries. J Am Heart Assoc. 10.1161/JAHA.117.007157 29275372 10.1161/JAHA.117.007157 PMC5779028

[48] Zhang DX, Mendoza SA, Bubolz AH, Mizuno A, Ge ZD, Li R, Warltier DC, Suzuki M, Gutterman DD (2009) Transient receptor potential vanilloid type 4-deficient mice exhibit impaired endothelium-dependent relaxation induced by acetylcholine in vitro and in vivo. Hypertension 53:532–538 19188524 10.1161/HYPERTENSIONAHA.108.127100 PMC2694062

[49] Garland CJ, Bagher P, Powell C, Ye X, Lemmey HAL, Borysova L, Dora KA (2017) Voltage-dependent Ca(2+) entry into smooth muscle during contraction promotes endothelium-mediated feedback vasodilation in arterioles. Sci Signal. 10.1126/scisignal.aal3806 28676489 10.1126/scisignal.aal3806

[50] Jackson WF (2000) Ion channels and vascular tone. Hypertension 35:173–178 10642294 10.1161/01.hyp.35.1.173 PMC1382026

[51] Touyz RM, Alves-Lopes R, Rios FJ, Camargo LL, Anagnostopoulou A, Arner A, Montezano AC (2018) Vascular smooth muscle contraction in hypertension. Cardiovasc Res 114:529–539 29394331 10.1093/cvr/cvy023 PMC5852517

[52] Brayden JE, Earley S, Nelson MT, Reading S (2008) Transient receptor potential (TRP) channels, vascular tone and autoregulation of cerebral blood flow. Clin Exp Pharmacol Physiol 35:1116–1120 18215190 10.1111/j.1440-1681.2007.04855.x PMC4193799

[53] Xia Y, Fu Z, Hu J, Huang C, Paudel O, Cai S, Liedtke W, Sham JS (2013) TRPV4 channel contributes to serotonin-induced pulmonary vasoconstriction and the enhanced vascular reactivity in chronic hypoxic pulmonary hypertension. Am J Physiol Cell Physiol 305:C704-715 23739180 10.1152/ajpcell.00099.2013 PMC3798671

[54] Shibasaki K, Ikenaka K, Tamalu F, Tominaga M, Ishizaki Y (2014) A novel subtype of astrocytes expressing TRPV4 (transient receptor potential vanilloid 4) regulates neuronal excitability via release of gliotransmitters. J Biol Chem 289:14470–14480 24737318 10.1074/jbc.M114.557132 PMC4031503

[55] Shibasaki K (2016) TRPV4 ion channel as important cell sensors. J Anesth 30:1014–1019 27506578 10.1007/s00540-016-2225-y

[56] Tureckova J, Hermanova Z, Marchetti V, Anderova M (2023) Astrocytic TRPV4 channels and their role in brain ischemia. Int J Mol Sci. 10.3390/ijms24087101 37108263 10.3390/ijms24087101 PMC10138480

[57] Ohashi K, Deyashiki A, Miyake T, Nagayasu K, Shibasaki K, Shirakawa H, Kaneko S (2018) TRPV4 is functionally expressed in oligodendrocyte precursor cells and increases their proliferation. Pflugers Arch 470:705–716 29569183 10.1007/s00424-018-2130-3

[58] De Logu F, Nassini R, Materazzi S, Carvalho Goncalves M, Nosi D, Rossi Degl’Innocenti D, Marone IM, Ferreira J, Li Puma S, Benemei S, Trevisan G, Monteiro S, de Araujo D, Patacchini R, Bunnett NW, Geppetti P (2017) Schwann cell TRPA1 mediates neuroinflammation that sustains macrophage-dependent neuropathic pain in mice. Nat Commun 8:1887 29192190 10.1038/s41467-017-01739-2 PMC5709495

[59] De Logu F, Geppetti P (2019) Ion channel pharmacology for pain modulation. Handb Exp Pharmacol 260:161–186 31820179 10.1007/164_2019_336

[60] Feng X, Takayama Y, Ohno N, Kanda H, Dai Y, Sokabe T, Tominaga M (2020) Increased TRPV4 expression in non-myelinating Schwann cells is associated with demyelination after sciatic nerve injury. Commun Biol 3:716 33247229 10.1038/s42003-020-01444-9 PMC7695724

[61] Matsumoto H, Sugio S, Seghers F, Krizaj D, Akiyama H, Ishizaki Y, Gailly P, Shibasaki K (2018) Retinal detachment-induced Muller glial cell swelling activates TRPV4 ion channels and triggers photoreceptor death at body temperature. J Neurosci 38:8745–8758 30143574 10.1523/JNEUROSCI.0897-18.2018 PMC6181316

[62] Machemer R (1968) Experimental retinal detachment in the owl monkey. II. Histology of retina and pigment epithelium. Am J Ophthalmol 66:396–410 4970986 10.1016/0002-9394(68)91523-7

[63] Machemer R, Norton EW (1969) Experimental retinal detachment and reattachment: I. Methods, clinical picture and histology. Bibl Ophthalmol 79:80–90 4981416

